# Supplementary material for: Comprehensive Annotation and Functional Exploration of MicroRNAs in Lettuce
Source: Front Plant Sci. 2021 Dec 24;12:781836. doi: 10.3389/fpls.2021.781836 (PMC8739914; doi:10.3389/fpls.2021.781836)
Supplement: Supplementary file 3 [file Data_Sheet_9.ZIP › GSM2230752.plot/Lsa-miR159b_Lsat_1_v5_gn_3_118460.1_3676_TPlot.pdf]

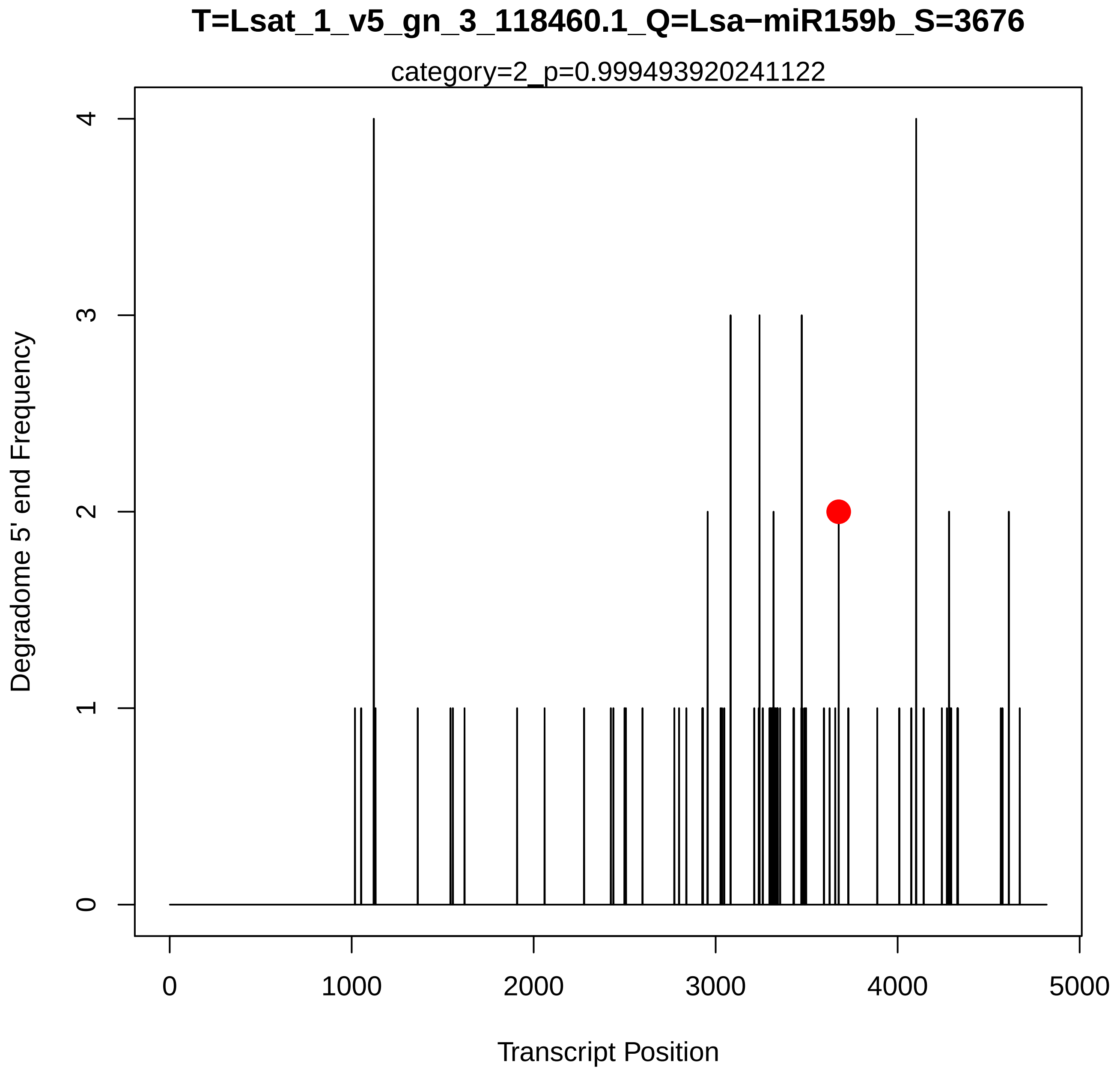

T=Lsat_1_v5_gn_3_118460.1_Q=Lsa−miR159b_S=3676
category=2_p=0.999493920241122
Degradome 5' end Frequency
0
1
2
3
4
0
1000
2000
3000
4000
5000
Transcript Position
